# Enhanced Serum Levels of sFlt1: Impact on Materno–Fetal CMV Transmission

**DOI:** 10.3390/jcm9051258

**Published:** 2020-04-26

**Authors:** Lukas Penka, Karl-Oliver Kagan, Klaus Hamprecht

**Affiliations:** 1 Institute for Medical Virology and Epidemiology of Viral Diseases, University Hospital of Tuebingen, Elfriede-Aulhorn-Straße 6, 72076 Tuebingen, Germany; 2 Department of Obstetrics and Gynaecology, University Hospital of Tuebingen, Calwerstrasse 7, 72076 Tuebingen, Germany

**Keywords:** CMV, pregnancy, soluble fms-like tyrosine kinase 1, sFlt1, placental growth factor, PlGF, hyperimmunoglobulin, HIG

## Abstract

Background: Antenatal Cytomegalovirus infection (CMV) can be associated with severe fetal symptoms and newborn outcome. The current prenatal diagnosis is based on amniocentesis (AC). No reliable biomarker for fetal infection is available. Methods: We measured Placenta-derived growth factor (PlGF), and soluble fms-like tyrosine kinase 1 (sFlt1), concentrations in maternal serum and amniotic fluid (AF) in context of maternal CMV primary infection. Blood sampling was carried out at the time of AC for detection of fetal CMV infection. The study cohort was divided into four subcohorts according to the presence or absence of fetal infection and preemptive hyperimmunoglobulin (HIG) treatment during the time interval between diagnosis of the CMV primary infection and AC. Results: The study cohort involved 114 pregnancies. In the non-transmitting subcohorts (NT) with and without prior HIG treatment, the median sFlt1 concentrations were 1.5 ng/mL (NT, HIG+) and 1.4 ng/mL (NT, HIG−), respectively. In the two transmitting groups (T) the concentrations were 1.3 ng/mL (T, HIG+) and 2.3 ng/mL (T, HIG−), respectively (NT, HIG− vs. T, HIG−, *p* < 0.001). The corresponding PlGF levels and the sFlt1/PlGF ratios showed no significant differences between the cohorts. The empirical cut-off values <1504 pg/mL sFlt1 and <307 pg/mL PlGF, were associated with the exclusion of CMV transmission (*p* < 0.001). Conclusion: sFlt1 concentration in the maternal blood could be a predictive biomarker for maternofetal CMV transmission.

## 1. Introduction

Maternal infection with the cytomegalovirus (CMV) belongs to the most relevant antenatal infections which can result in the severe physical and mental developmental disorder of the offspring. The cases with the most severe postnatal sequelae are usually due to maternal infections in the first trimester where materno–fetal transmission occurs prior to 20–23 weeks of gestation. Later on in the pregnancy, the materno–fetal transmission rate is higher but hardly any child will be seriously symptomatic after birth.

To date, the diagnosis of a fetal infection can only be reliably made by amniocentesis (AC) [1]. Although invasive testing is relatively safe, it still carries a risk of miscarriage in about 1 in 1000 procedures. Therefore, current research focuses on ultrasound and biomarkers that could be used as surrogate parameters.

In the previous case study of Oliveira et al., the serum levels of soluble fms-like tyrosine kinase 1 (sFlt1) and placental growth factor (PlGF) were altered during maternal CMV infection [2]. The authors linked their observations with histological findings in placental tissue, such as a reduced number of chorionic villi and blood vessels. Abnormal placental microarchitecture, reduced blood flow, hypoxia and subsequent inflammatory response are well known in the pathogenesis of a symptomatic fetal CMV infection [3].

PlGF is a member of the vascular endothelial growth factor (VEGF) family involved in neoangiogenesis. It is synthesized in dependency on oxygen tension and changes over the course of the pregnancy. PlGF binds VEGF-receptor (Flt1), a receptor tyrosine kinase. Its soluble splice variant sFlt1 binds free PlGF and therefore antagonizes its effects [3]. The two serum markers sFlt1 and PlGF, especially the ratio of both, is generally used to stratify the individual risk of preeclampsia (PE) in symptomatic women in the context of the imbalance between angiogenic factors such as vascular endothelial growth factor (VEGF) or PlGF and anti-angiogenic factors like sFlt1 [4]. PE is defined as a new-onset hypertension in pregnancy accompanied by proteinuria after 20 weeks of gestation. Its pathophysiology as far as known has been described as a strong inflammatory reaction in uterine vessels [5] with an increase in CD4+ T-cells and inflammatory cytokines, along with an increase in reactive oxygen species and sFlt1 [6]. Early-onset PE developing before 34 weeks of gestation is related to an incomplete trophoblast invasion and a failure of normal spiral artery remodeling. sFlt1 is capable of binding and reducing PlGF levels, which leads to reduced proangiogenic signaling and insufficient spiral artery remodeling [7].

In this study, we analyzed the maternal serum marker levels of sFlt1 and PlGF at the time of AC due to maternal primary CMV infections. Furthermore, we compared the serum levels of the two markers with corresponding concentrations in the amniotic cavity.

## 2. Material and Methods

The department of prenatal medicine at the University of Tuebingen, Germany, is a national tertiary referral center with a special interest in the management of primary CMV infection in pregnancy. When women were referred in the first trimester due to a recent primary CMV infection, we administered 200 IU per kg body weight of hyperimmunoglobulins (HIG) every 2 weeks for up to 20 weeks. A detailed description of the inclusion criteria is given elsewhere [8,9]. In short, for inclusion in the HIG treatment protocol, the diagnosis of a primary CMV infection should be made up to 14 + 6 weeks. Furthermore, the CMV IgG level (ECLIA) should be < 60 U/mL, reactivity against gB2 should not be detectable or just at onset of seroconversion (score 0 or 1, recIB), the CMV IgG avidity index (AI) should be < 45% (ECLIA) and the IgM index (ECLIA) should be > 1.0. In all other conditions, we followed the international recommendation [10,11] and did not administer HIG treatment. Prior to the implementation of our strict HIG administration protocol in 2013, we also administered HIG treatment to second trimester primary CMV infections, but with limited success.

In all cases with a proven first and early second trimester primary infection or in cases with anomalies that indicate a primary CMV infection [9,12], an AC is carried out at least six weeks after the assumed infection, predominantly at 20–23 weeks gestation. The viral transmission state is examined by using short-term (18 h-) microculture, viral isolation, in-house nested PCR (UL123/IE1-Ex4) and quantitative-rtPCR (UL83/pp65), respectively [8].

At the time of the AC, we also drew maternal blood to assess the IgG and IgM levels as well as the IgG avidity (ECLIA). After the analysis is finalized the blood and the amniotic fluid (AF) was cryopreserved at −80 degree Celsius. None of the pregnancies were complicated by preeclampsia.

For this retrospective study, we used the cryopreserved samples to measure PlGF and sFlt1 concentrations in the maternal serum and in the AF. For the analysis, we used the Elecsys PlGF- and sFlt1-ECLIA-assays on a Cobas 6000 analyzer (Roche, Mannheim, Germany). Both test systems are approved automated routine tests in preeclampsia diagnosis. In cases when sFlt1 concentrations in AF exceeded the measuring range, AF was diluted tenfold for processing. 

The institutional ethics board approved this study (University Hospital of Tuebingen: project 237/2019BO2).

Statistical analysis: The pregnancies were stratified into four subcohorts according to whether HIG treatment was administered or not (HIG + or −) and according to the transmission status at the time of AC (T = transmitter, NT = non transmitter). The results are presented as median (25–75th interquartile range: IQR). The statistical analyses were performed using SPSS V.23 (IBM, Armonk, NY, USA). Significant differences were assumed at a *p* level of 0.05.

Ten women were latently CMV-infected at first diagnosis and served as a control. In 72 out of these 114 cases, simultaneously collected AF samples were evaluated. Additionally, we stratified additional cohorts by the presence of ultrasound abnormalities associated with a CMV infection. We used receiver operating characteristic (ROC) analyses for evaluation of the potential cut-off values. 

## 3. Results

We evaluated 114 serum samples from 114 pregnant women for PlGF and sFlt1 concentrations. In 34 cases, we observed materno–fetal transmission at the time of AC. Of those, 18 women did not receive HIG treatment (group A: T and HIG−) and in 16 cases, HIG treatment was administered (group B: T and HIG+). Of note, only three women in the latter group were enrolled in the ongoing Tuebingen HIG study [8]. In 80 pregnancies, materno–fetal transmission did not occur. Twenty-one women did not receive HIG treatment (group C: NT and HIG−) and 49 did (group D: NT and HIG). Ten women were latently CMV-infected at the first diagnosis and served as a control. Further study details are given in Table 1.

### 3.1. Serum-Analysis of Subcohorts

**Figure 1:**
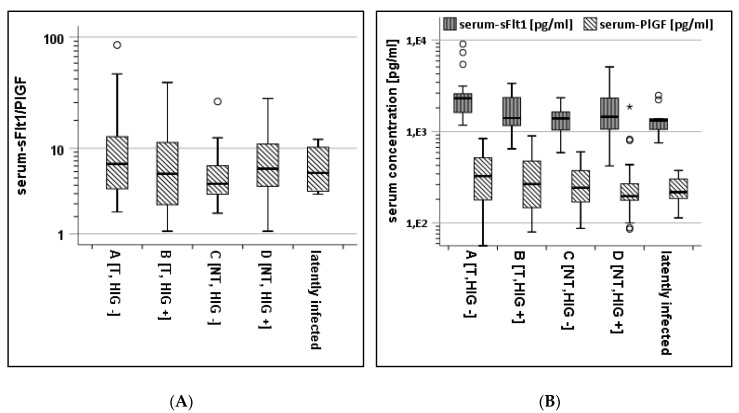
(**A**).Serum sFlt1/PLGF-ratio of NT and T −/+ HIG and latently infected women. (**B**). Serum PlGF and sFlt1 concentrations of NT and T −/+ HIG. * means >3 SD. °, *: outliers and extreme outliers, respectively.

Figure 1A shows the sFlt1/PlGF ratios in the subcohorts A to D as well as in the latently infected pregnant mothers. There was no significant difference in the sFlt1/PlGF ratio between the four subcohorts (*p* = 0.27 in the Kruskal–Wallis test). Figure 1B shows the absolute serum concentrations for both proteins. Highly significant differences were shown with higher sFlt1 concentrations in cohort A, and good selectivity was shown between NT and T in HIG-untreated women (A, C). This selectivity is not visible in the subcohort analyses of PlGF values although PlGF concentrations are high in the subcohort of HIG-naïve transmitters (A).

Table 2 summarizes the measurements in each subcohort. The median sFlt1 concentration in the serum was the highest in subcohort A (T/HIG−) with 2.3 ng/mL (IQR: 1.1), while the median sFlt1 serum concentrations in all other subcohorts were lower with 1.4 ng/mL (B), 1.4 ng/mL (C) and 1.5 ng/mL (D); *p* = 0.01 in the Kruskal–Wallis test (Figure 1B). Comparing cohorts A and C reveals highly significant differences; *p* < 0.001 in the Mann–Whitney test. There were no significant differences in the PlGF levels (*p* = 0.24 in Kruskal–Wallis test).

### 3.2. AF-Analysis of Subcohorts

Table 3 gives the median marker levels in the AF and the respective ratio. The Kruskal–Wallis test showed a significant difference for both markers, sFlt1 (*p* < 0.01) and PlGF (*p* < 0.05) and the Mann–Whitney test demonstrated a highly significant difference between cohorts A and D for both, sFlt1 (*p* < 0.001) and PlGF (*p* < 0.01).

### 3.3. Protein-Levels and Ultrasound Examinations

**Figure 2:**
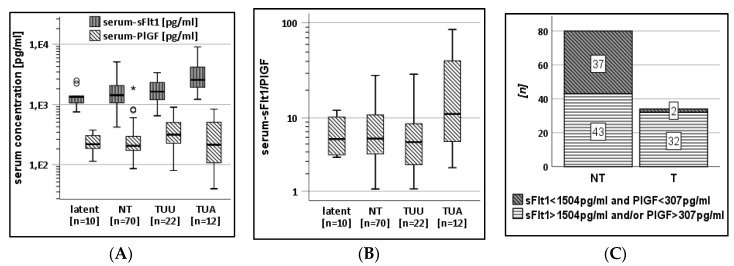
(**A**). Serum sFlt1 and PlGF concentrations in subcohorts divided by non-transmitters, transmitters without ultrasound unsuspicious for CMV (T_UU_) compared with latently infected, and a subcohort of women with ultrasound abnormalities (T_UA_) associated with fetal CMV infection. (**B**). Serum sFlt1/PlGF ratio in subcohorts according to transmission status and ultrasound findings revealing significant differences between the subcohort T_UA_ compared with T_UU_ and NT, respectively. (**C**). Frequency of samples below and above empirical cut-off values for PlGF and sFlt1 separated by transmission status at AC. Transmission is more frequently associated with sFlt1 or PlGF concentrations above 1504 pg/mL and 307 pg/mL, respectively. °, *: outliers and extreme outliers, respectively.

For this analysis, the following subcohorts were analyzed: latently infected pregnant mothers (latent), maternal non-transmitters (NT), transmitters with unsuspicious ultrasound (T_UU_), versus transmitters with ultrasound abnormalities (T_UA_). In 12 of 34 cases of CMV transmission, the fetal ultrasound showed abnormalities (T_UA_) linked to CMV infection at around 20 weeks GA. Three of 12 women from the T_UA_-subcohort received HIG prior to AC. Within the subcohort of women with an unsuspicious ultrasound (T_UU_), 13/22 women received HIG treatment. The serum sFlt1 concentrations were over 1.5 fold increased ($\tilde{x}$ = 2.6 ng/mL; IQR:3.1) in the T_UA_-subcohort compared to T_UU_ ($\tilde{x}$ = 1.6 ng/mL; IQR:1.1; *p* < 0.01 in the Mann–Whitney test). In contrast, the median PlGF values were higher in the T_UU_-subcohort (315 pg/mL; IQR:279) compared to T_UA_ with 217 pg/mL (IQR: 406; *p* = 0.33 in the Mann–Whitney test) and the non-transmitting subcohort ($\tilde{x}$ = 205 g/mL; IQR:126; *p* < 0.05 in the Mann–Whitney test; Figure 2A). Figure 2B shows the sFlt1/PlGF ratio in subcohorts arranged by ultrasound findings. The median sFlt1/PlGF ratio in the T_UA_-subcohort ($\tilde{x}$ = 11; IQR:36) was significantly higher with a large range compared to the T_UU_-subcohort ($\tilde{x}$ = 5; IQR:6; *p* < 0.02 in the Mann–Whitney test) and the NT-subcohort ($\tilde{x}$ = 6; IQR:7; *p* < 0.04 in the Mann–Whitney test).

### 3.4. ROC Analyses

In ROC analysis for the detection of transmission with sFlt, PlGF, and the ratio, the area under the curve (AUC) was 0.66 (95%CI 0.55–0.77; *p* < *0*.01), 0.61 (95%CI 0.48–0.74; *p* = 0.07) and 0.52 (95% CI 0.39–0.65; *p* = 0.73), respectively. Predicting ultrasonographical symptomatic fetal infection by serum protein concentrations results in an 0.82 AUC 95%CI (0.69, 0.94); *p* < *0*.001 for sFlt1 and 0.462 AUC 95%CI (0.24, 0.69); *p* = 0.66 for PlGF (Figures S1–S6).

The ROC-derived cut-off values, <1504 pg/mL for sFlt1 and <307 pg/mL for PlGF, correctly predict non-transmission at AC in 37/39 cases with concentrations below these cut-off values. The frequency of both protein concentrations below these cut-offs is highly significant for non-transmitters (*p* < 0.001 in Fisher’s exact test; Figure 2B).

## 4. Discussion

Prenatal diagnosis of vertical CMV transmission is based on invasive AC. Fetal risk evaluation might be performed from fetal blood after cordocentesis. There is a need for a less invasive biomarker-based diagnosis. This study retrospectively evaluates serum and AF levels of sFlt1 and PlGF under the influence of fetal CMV infection and HIG treatment for prevention of diaplacental transmission.

After AC, 114 serum and 72 AF samples were categorised by HIG treatment and transmission state. This led to four subcohorts (A–D) showing that AF from women after CMV transmission without prior HIG treatment contains higher levels of PlGF and its soluble receptor sFlt1 compared to non-transmitting women with or without HIG treatment, discordant to published data. Maidji et al. showed the increase in sFlt1, but not PlGF, in AF leading to an increase in the sFlt1/P1GF ratio comparing pregnant women after HIG treatment and untreated women in 47 cases of congenital CMV infection with seven seronegative controls [3]. Our data document for the first time that highly significant differences between sFlt1 levels in CMV-transmitting and non-transmitting women are also observable in serum prior to AC, as shown for cohorts A and C (Figure 1B). The sFlt1/PlGF ratio was not altered by transmission although some cases of transmission were associated with elevated values >38 (Figure 1A). If prenatal ultrasound showed signs associated with CMV-infection of the fetus, the sFlt1/PlGF ratio in transmitting women was significantly higher compared to women transmitting without suspicious ultrasound examinations and compared to non-transmitting women (Figure 2B).

The biomarkers for the prediction of diaplacental transmission have a very high clinical relevance. Assuming an increase in sFlt1 concentration after CMV transmission resulted in an AUC of 0.66 in ROC analyses. Two individual cut-off values for both proteins showed a highly significant (*p* < 0.001) association with non-transmission with only two of 34 CMV-transmitting women showing values below these cut-offs (Figure 2B). Nonetheless, a large proportion of non-transmitting women (43/80) also showed sFlt1 and/or PlGF concentrations above these values (Figure 2C). The potential of these two cut-offs is clearly in exclusion of transmission rather than its diagnosis. Under these conditions, women with concentrations above the presented cut-offs would need further diagnostic methods for the exclusion of transmission.

A prospective evaluation of sFlt1 and PlGF concentrations for the exclusion or prediction of CMV transmission might be an approach for a less invasive diagnosis of fetal CMV infection. Using the two proteins with evaluated cut-off values from larger cohorts might potentially decrease the need and frequency for invasive AC, that, in many cases, produces anxiety [13], and potentially cause complications as well as the unnecessary termination of pregnancy.

To date, no efficient tool is established for individual risk evaluation for diaplacental CMV transmission or the diagnosis of fetal infection except for virus detection from AF following AC or postnatal body fluids [1]. This lacks evidence for the prenatal prediction of postnatal outcome of the child. Fetal platelet counts, increased viral load or anti-CMV-IgM, were described as indicators for poor outcome, like clinical symptoms at birth or histological findings in placentae [14,15]. β2-microglobulin levels in fetal blood are a possible biomarker for the differentiation between symptomatically and asymptomatically infected fetuses [15]. All these invasive approaches need fetal blood sampling.

Guerra et al. showed that ultrasound abnormalities can be used for the determination of symptomatic infection with a positive predictive value of 35% in all fetuses following maternal CMV-PI, and 78% in the subcohort of diaplacentally infected fetuses [16]. This needs further research to evaluate whether sFlt1 and PlGF allow for the prediction of symptomatic postnatal CMV infection. Our observation that CMV-linked ultrasound abnormalities are frequently combined with increased maternal sFlt1 serum concentrations (Figure 2A) might open the door for future studies with larger, more strictly defined cohorts and prospective study design, linking prenatal biomarker levels with postnatal symptoms and outcome.

The HIG-treated subcohort B showed sFlt1 and PlGF concentrations comparable with healthy non-transmitters in serum (Figure 1B). This might indicate a reduced inflammation after passive immunization. Interestingly, both protein concentrations in AF were correlating in our cohort (Figure S7). One reason for an increase in both proteins might be the extent of placental damage due to viral replication. The current literature repeatedly shows an association of high sFlt1 values with lower PlGF concentrations [17,18]. We saw this effect in some cases after CMV transmission and suspicious fetal ultrasound (Figure 2A).

In conclusion, we documented for the first time the potential predictive value of sFlt1 and, subordinated PlGF, in serum as surrogate markers for maternofetal CMV transmission by defining cut-off-levels for both proteins for the non-invasive diagnosis of fetal CMV infection. Our systematical, retrospective analysis of defined subcohorts of maternal CMV transmitters and non-transmitters and those with CMV-related ultrasound abnormalities confirms two initial case reports observing increased levels of circulating antiangiogenic sFlt1 during CMV primary infections in pregnancy. However, in contrast to these initial observations our study cohort did not express a CMV-induced mirror syndrome in the context of PE [2,19]. Further longitudinal and prospective analysis of sFlt1 and PlGF concentrations in serum during first trimenon will be able to evaluate the predictive value of sFlt1 increase as a biomarker for CMV transmission. Limitations and perspective: The results of our study are limited due to a restricted case number (*n* = 114), especially in the CMV-transmitting subcohort which included only 34 women. However, our data clearly show the potential benefits of serum sFlt1 and PlGF levels analysis for mothers with CMV-PI. Prospective, multicenter studies with larger cohorts for the proof of concept using PlGF and sFlt1 are needed. Based on larger cohorts, the evaluation of antenatal maternal serum sFlt1 and PlGF levels together with the postnatal outcome could point towards suitable biomarkers. HIG treatment as a prevention strategy for diaplacental CMV infection is safe and effective for women with CMV-PI [8,20,21]. Currently, the number of women treated preventatively with biweekly, high-dose HIG following CMV-PI is increasing in our facility, although initial CMV screening in pregnancy is not covered by German health insurers and the diagnosis of CMV-PI mainly happens accidentally. Therefore, the need for early non-invasive and reliable methods for the prediction of materno–fetal transmission is urgent.

## Figures and Tables

**Table 1:** Subcohort characteristics stratified by treatment with HIG and CMV transmission status.

Characteristics	T, HIG−	T, HIG+	NT, HIG−	NT, HIG+	
sample size	18	16	21	49	
maternal age at PI, years, mean (SD)	31 (6)	32 (4)	32 (4)	30 (4)	n.s.^¶^
gravidity, median (IQR)	2 (2)	2 (1)	2 (1)	2 (2)	n.s.^§^
parity, median (IQR)	1 (1)	1 (0)	1 (0)	1 (1)	n.s.^§^
GA at amniocentesis, median (IQR)	22 (5)	21 (2)	22 (5)	20 (1)	n.s.^§^
HIG applications, mean (SD)		3 (2)		4 (1)	n.s.^#^
bodyweight, kg, median (IQR)		65 (23)		63 (13)	n.s.^&^

HIG: hyperimmunoglobulin, T: CMV transmission, NT: CMV non-transmission, PI: CMV primary infection, SD: standard deviation, IQR: interquartile range, GA: gestational age, ¶ ANOVA, § Kruskal–Wallis test, # *t*-test, & Mann–Whitney test.

**Table 2:** Serum protein concentrations of sFlt1 and PlGF and sFlt1/PlGF ratio in subcohorts.

Characteristics	T, HIG− (A)	T, HIG+ (B)	NT, HIG− (C)	NT, HIG+ (D)	Latent
Sample size	18	16	21	49	10
Median sFlt1 in ng/mL (IQR)	2.3 (1.1)	1.4 (1.2)	1.4 (0.7)	1.5 (1.3)	1.3 (0.6)
Median PlGF in pg/mL (IQR)	326 (363)	268 (345)	243 (211)	197 (104)	218 (130)
Median sFlt1/PlGF (IQR)	7.1 (9.2)	5.6 (9.4)	4.4 (3.7)	6.3 (7.2)	5.8 (6.8)

sFlt1: soluble fms-like tyrosine kinase 1, PlGF: placental growth factor, IQR: interquartile range.

**Table 3:** Amniotic fluid protein concentrations of sFlt1 and PlGF and the sFlt1/PlGF ratio.

Characteristics	T, HIG− (A)	T, HIG+ (B)	NT, HIG− (C)	NT, HIG+ (D)	Latent
Sample size	13	11	9	31	8
Median sFlt1 in ng/mL (IQR)	99 (139)	63 (117)	67 (109)	54 (49)	39 (47)
Median PlGF in pg/mL (IQR)	239 (201)	172 (190)	214 (158)	173 (92)	125 (96)
Median sFlt1/PlGF (IQR)	423 (265)	438 (316)	492 (245)	355 (245)	291 (183)

sFlt1: soluble fms-like tyrosine kinase 1, PlGF: placental growth factor, IQR: interquartile range.

## Supplementary Materials

The following figures are available online at https://www.mdpi.com/2077-0383/9/5/1258/s1, Figure S1: ROC-Curve for sFlt1 and transmission state, Figure S2: ROC-Curve for PlGF and transmission state, Figure S3: ROC-Curve for sFlt1/PlGF and transmission state, Figure S4: ROC-Curve for sFlt1 and ultrasound abnormalities linked to CMV-infection, Figure S5: ROC-Curve for PlGF and ultrasound abnormalities linked to CMV-infection, Figure S6: ROC-Curve for sFlt1/PlGF and ultrasound abnormalities linked to CMV-infection, Figure S7: Amniotic fluid concentrations are correlating with Spearman’s ρ = 0.73.
