# Supplementary material for: Enhanced Serum Levels of sFlt1: Impact on Materno–Fetal CMV Transmission
Source: J Clin Med. 2020 Apr 26;9(5):1258. doi: 10.3390/jcm9051258 (PMC7287861; doi:10.3390/jcm9051258)

Supplemental materials

Figure S1. ROC-Curve for sFlt1 and transmission state.

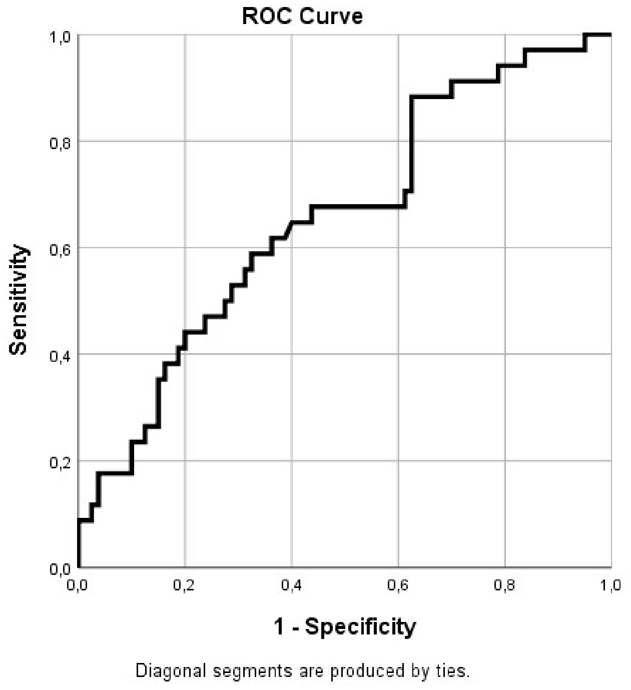

AUC: .656; 95% CI [.55 to .77]; *p*<.01

Figure S2: ROC-Curve for PlGF and transmission state

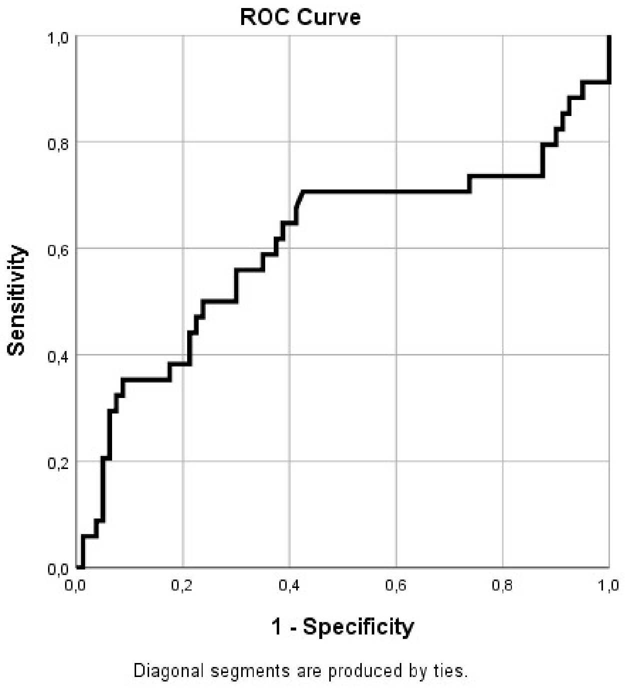

AUC: .606; 95% CI [.48 to .74]; *p*=.07

Figure S3: ROC-Curve for sFlt1/PlGF and transmission state

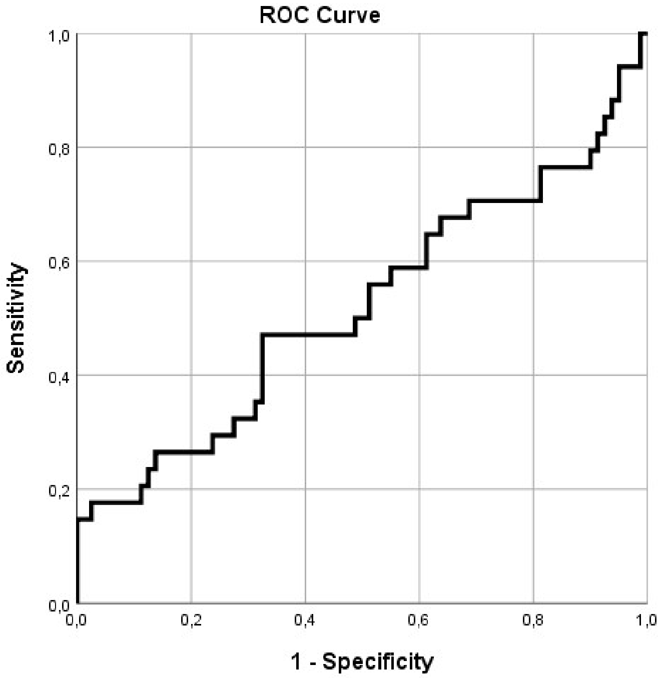

AUC: .52; 95% CI [.39 to .65]; ***p*=.73**

Figure S4: ROC-Curve for sFlt1 and ultrasound abnormalities linked to HCMV-infection

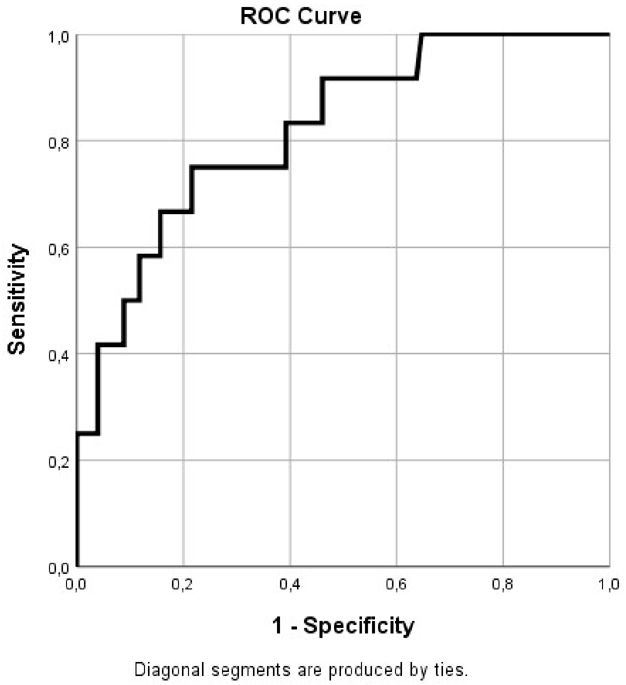

AUC: .821; 95% CI [.69 to .94]; ***p*<.001**

Figure S5: ROC-Curve for PlGF and ultrasound abnormalities linked to HCMV-infection

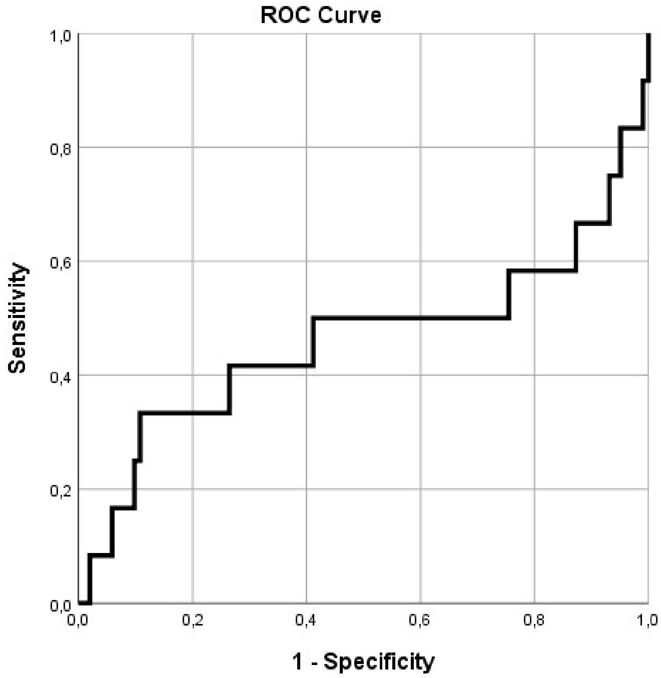

AUC: .462; 95% CI [.24 to .69]; *p*=.66

Figure S6: ROC-Curve for sFlt1/PlGF and ultrasound abnormalities linked to HCMV-infection

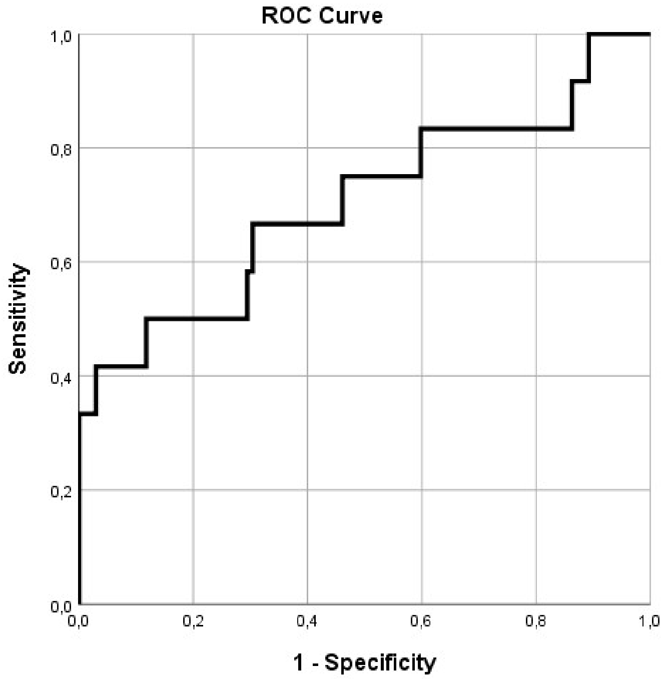

AUC: .703; 95% CI [.52 to .89]; *p*<.05

Figure S7. Amniotic fluid concentrations are correlating with Spearman's ϱ = .73.

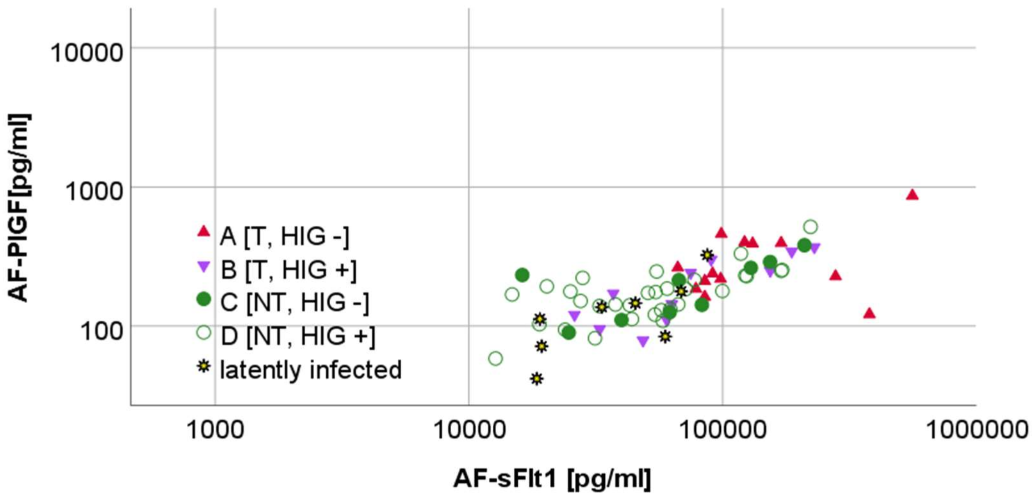

Supplement: Supplementary file 1 [file jcm-09-01258-s001.pdf]
